# Experimental model to evaluate the benefits of lutein to prevent retinal phototoxicity during pars plana vitrectomy surgery using xenon source light illumination in rabbits

**DOI:** 10.1186/s40942-019-0161-3

**Published:** 2019-05-07

**Authors:** Anderson Teixeira, Eduardo A. Novais, Emmerson Badaró, Acácio Lima, Michel Eid Farah, Rubens Belfort

**Affiliations:** Departamento de Oftalmologia, Universidade Federal de São Paulo, Secretaria Administrativa, Rua Botucatu, 821, 1o andar, São Paulo, SP 04023-062 Brazil

**Keywords:** Lutein, Photo toxicity, Xenon light, Pars plana vitrectomy

## Abstract

**Background:**

To evaluate the benefits of lutein in preventing retinal phototoxicity generated by xenon light sources during vitreoretinal surgery.

**Methods:**

A prospective cross-sectional study in pigmented rabbit eyes exposed to different vitreoretinal surgery lighting simulations. Twenty Dutch-belted rabbits were divided into two groups exposed to two different xenon wavelength light sources filters (420 nm and 435 nm). In addition, two subgroups were administered with daily supplemental of 10 mg of Lutein systemically. Electroretinography (ERG), optical coherence tomography (OCT) and fluorescein angiography (FA) were performed before and after surgery to quantify the retinal damage.

**Results:**

All animals submitted to the experiment presented some degree of phototoxicity independent of wavelength light filter used. Retinal damage was evident as the FA presented areas of hyperfluorescence, and the OCT depicted increased reflective areas of the inner and outer retinal layers, and RPE. ERG showed a diffuse reduction of the a and b waves amplitudes in all animals.

**Conclusion:**

Use of systemic administration of lutein showed no benefit to avoiding retinal phototoxicity generate to xenon light source using filters of 435 nm and 420 nm when comparing to the control group.

## Background

The eye is constantly exposed to sunlight and artificial light, which is essential for its biological functions such as vision and regulation of the circadian rhythm. However, when the exposure to light is excessive, radiation may generate visual impairment and even blindness [1].

**Fig. 1:**
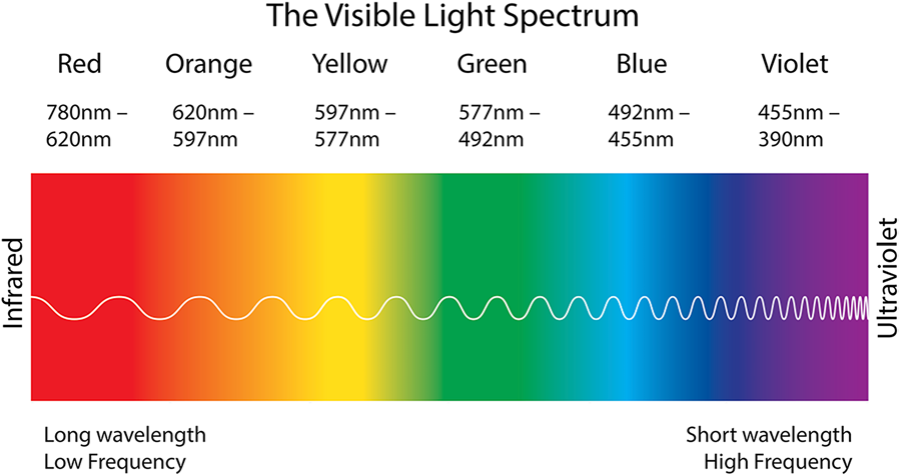
Visible light spectrum. *Source* www.Google.com

In vitreoretinal surgery, the use of an intraocular illumination is necessary, increasing light exposure to the vitreous, retina and subretinal space [2]. Four types of light sources are currently available: Metal halide, xenon, mercury, and halogen [3]. The use of light sources, however, can cause photochemical and thermal damage as an adverse effect to the retina [4]. The photochemical toxicity occurs when the eye is exposed to radiation light from the lowest visible wavelength (blue and violet), which becomes more harmful with wavelengths between 380 and 550 nm (Fig. 1) [5]. The thermal damage is caused by light sources with high-intesity irradiation/irradiance such as laser or xenon, causing the heating of tissues that absorb the radiation [6]. The retinal pigment epithelium (RPE) is prone to this type of injury due to the increased amount of pigment that can absorb light from different wavelengths.

Lutein and Zeaxanthin are carotenoids present in the macular retinal pigment. A study in primates using microdensitometry observed a large pigment density in the axons of the photoreceptors (outer plexiform layer) in the foveal area and adjacent layers (inner and outer plexiform). The macular pigment also functions as a filter that absorbs short wavelengths of visible light, reducing chromatic aberration and dispersion in the. Filtration of short wave helps in preventing photochemical damage to cones and EPR [7–10].

In this study, we evaluated the benefits of supplemental lutein in preventing retinal phototoxicity generated by light sources for vitreoretinal surgery simulations in pigmented rabbits using two wavelengths (420 nm and 435 nm).

## Materials and methods

The study was conducted in the Retina and Vitreous experimental laboratory of the Department of Ophthalmology of the Federal University of São Paulo. Twenty-Dutch belted rabbits weighing 1.5–2 kg were used according to the research standards of the Declaration of Helsinki and the Association for Research in Vision and Ophthalmology (ARVO) for experiments on animals.

A single experimental procedure was performed on the right eye of each rabbit. In total, 20 animals were divided into four groups (five animals for each group). They were divided into two groups exposed to different wavelengths (420 nm and 435 nm) xenon lights (Xenon BrightStar illumination, DORC Inc, Netherlands) sources. The two wavelengths were chosen for this study as the 420 nm is the cut-off for core vitrectomy and general membrane removal, and 435 nm for removal of membranes adherent to the retina. In addition, two subgroups received supplemental 10 mg of Lutein systemically mixed in the feed (LUT 10, Ophthalmos Inc, Brazil) and exposed to 420 nm or 435 nm wavelength. All rabbits were submitted to a macular surgery simulation during the experimental procedure, with the endoillumination placed near the retina with 30 min of light exposure. The maximum intensity of each of the light sources and endoillumination probe did not exceed the commercially used and no microscope light was used during the experiment.

### Data acquisition

All rabbits were submitted to Electroretinography (ERG), optical coherence tomography (OCT) and fluorescein angiography (FA) preoperatively and seventh postoperative day.

### Anesthesia and preoperative preparation

All surgical procedures and tests were performed under anesthesia after intramuscular injection of 35 mg/kg of ketamine hydrochloride (Phoenix Scientific Inc., USA) and 5 mg/kg of xylazine hydrochloride (Phoenix Scientific Inc., USA) in the cranial thigh (quadriceps) muscles. The pupil was dilated by instillation of cyclopentolate hydrochloride 1% (Bausch & Lomb Pharmaceuticals Inc., USA) and 5% phenylephrine (Bausch & Lomb Pharmaceuticals Inc., USA).

### Surgical technique

The animals were covered with sterile drapes and an eyelid speculum placed, followed by instillation of a drop of 5% povidone-iodine. The following procedures were performed: 25 gauge sclerotomy for insertion of the light probe and placed on a fixed position using a micromanipulator. At the end of the procedure, the sclerotomy sight was sutured using 7-0 Vycril, and a sterile drop of antibiotics and steroids was applied. The rabbits were sacrificed by intravenous injection of 2 ml of phenobarbital seven days after surgery.

### Electroretinography

ERG was performed preoperatively and seven days after the procedure. Rabbits were kept in a dark room for one hour before the exam. Dark red illumination was used to handle the rabbits as they were anesthetized with an intramuscular injection of ketamine and xylazine. After dilation of the pupils, an electrode (Burian Allen, Hansen Ophthalmic, Iowa City, USA) covered with 1% methylcellulose was placed on the surface of the cornea; another similar electrode was placed on the temporal side as reference electrode; a gold dome of ground electrode in clip form, filled with electrolytic gel was placed in the animal’s ear.

Dark-adapted ERG “a” (rod-mediated) and “b”-waves (mediated by bipolar and Müller cells) evoked at a luminance of 1.30 log cd s/m 2 were recorded. The responses from five consecutive flashes generated an average to determine the dark adaptation responses. After light adaptation for at least 25 min, light-adapted ERG was performed, and “a”-wave (cone-mediated) evoked at a luminance of 1.30 log cd s/m 2 were recorded. The ERG followed the standard protocol recommended by ISCEV (International Society for Clinical Electrophysiology of Vision), in which five responses are recorded: (1) rods scotopic response; (2) maximum scotopic response (rods and cones); (3) scotopic response of oscillatory potentials; (4) photopic response of cones to single flash; (5) photopic flicker response to 30 Hz. The parameters analyzed were the amplitudes of the responses, measured in microvolts (µV) and the implicit time of b-wave, measured in milliseconds (ms). All values were compared with standard values using the same procedure standardized in our laboratory.

### Retinography

The color fundus photography was performed using a specific fundus camera (TRC Topcon, Topcon, Tokyo, Japan) preoperatively and seven days after the procedure (depending on the subgroup).

### Fluorescein angiography (FA)

FA was performed by intravenous injection of 0.3 ml of sodium fluorescein 10% (Ophthalmos, São Paulo, Brazil) in the auricular vein of the animal. FA was performed preoperatively and seven days after the procedure using HRA angiography (Heidelberg Inc, Germany).

### Optical coherence tomography (OCT)

OCT was performed preoperatively and seven days after the procedure. This examination was conducted using the spectral-domain Spectralis OCT (Heidelberg Inc, Germany). After pupillary dilation, a 20 × 15-degree high-resolution 19 sections B-scan spaced by 120 µm acquisition protocol was performed at the inferiorlly, inferotemporally and inferonasally to the optic nerve.

### Analysis of results

The analysis of the results was carried out by qualitative and quantitative comparison of the preoperative and postoperative ERG, OCT and FA.

## Results

**Fig. 2:**
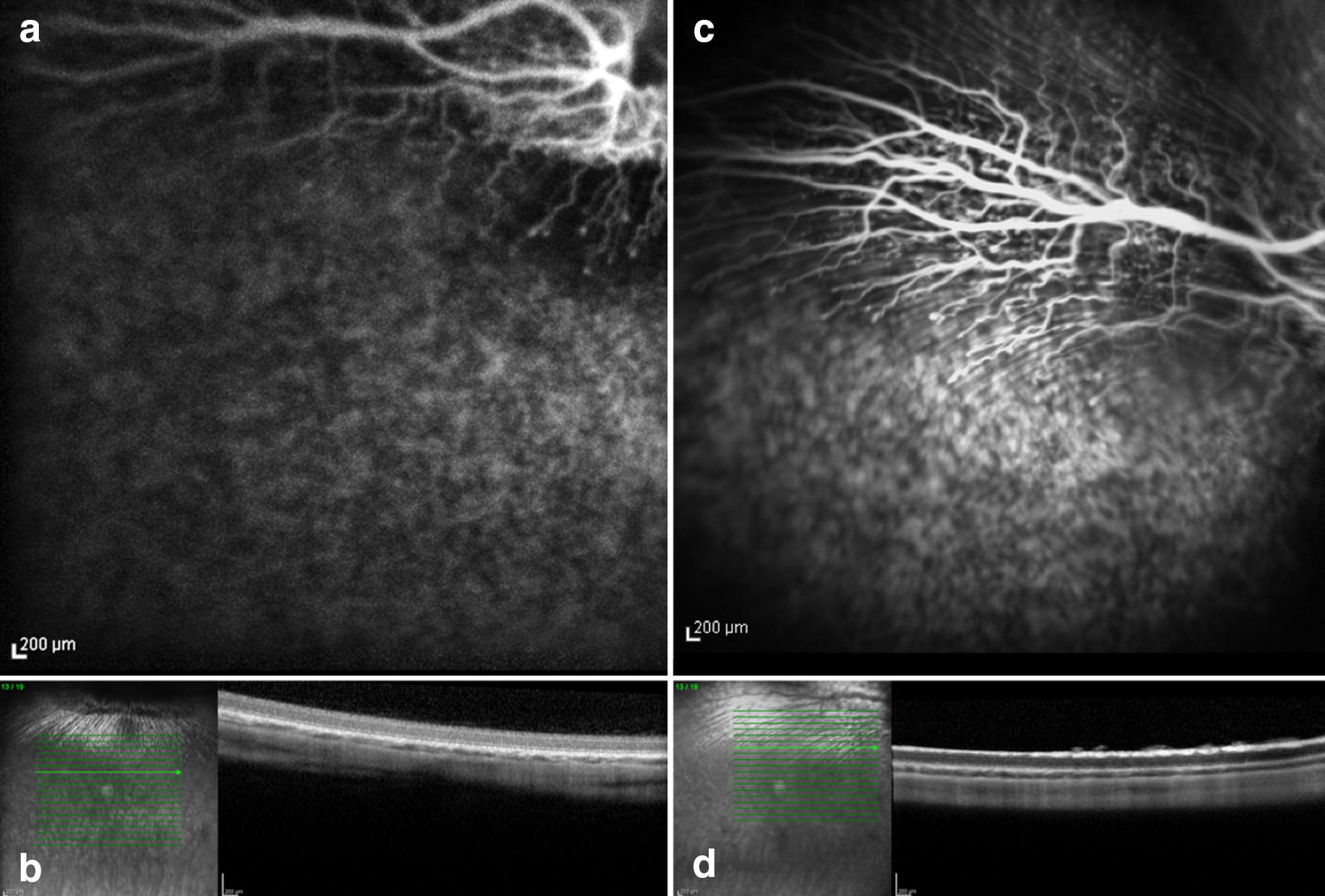
Preoperative and seven days posoperative fluorescein angiography and OCT. Rabbit (no. 1) exposed to 420 nm illumination after systemic administration of lutein. Postoperative hyperfluorescence corresponds to the exposed area and increased reflective of the inner and outer layers of the retina seen on OCT

**Fig. 3:**
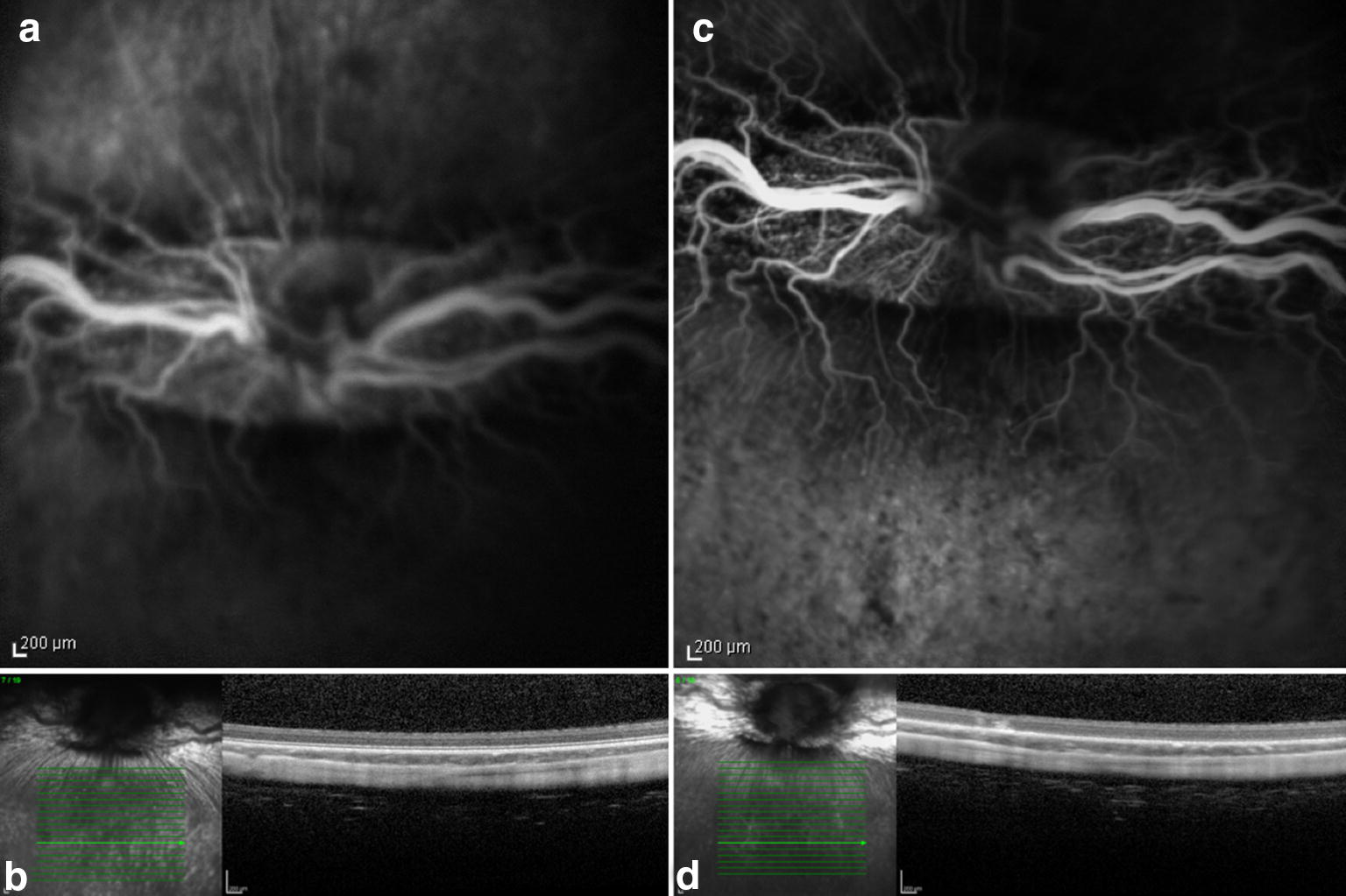
Preoperative (**a** and **b**) and seven days postoperative (**c** and **d**) fluorescein angiography and OCT. Rabbit (no. 2) exposed to light of 420 nm after systemic administration of lutein. Postoperative hyperfluorescence (**c**) corresponds to the exposed area and increased reflective of the inner and outer layers of the retinaseen on OCT (**d**)

**Fig. 4:**
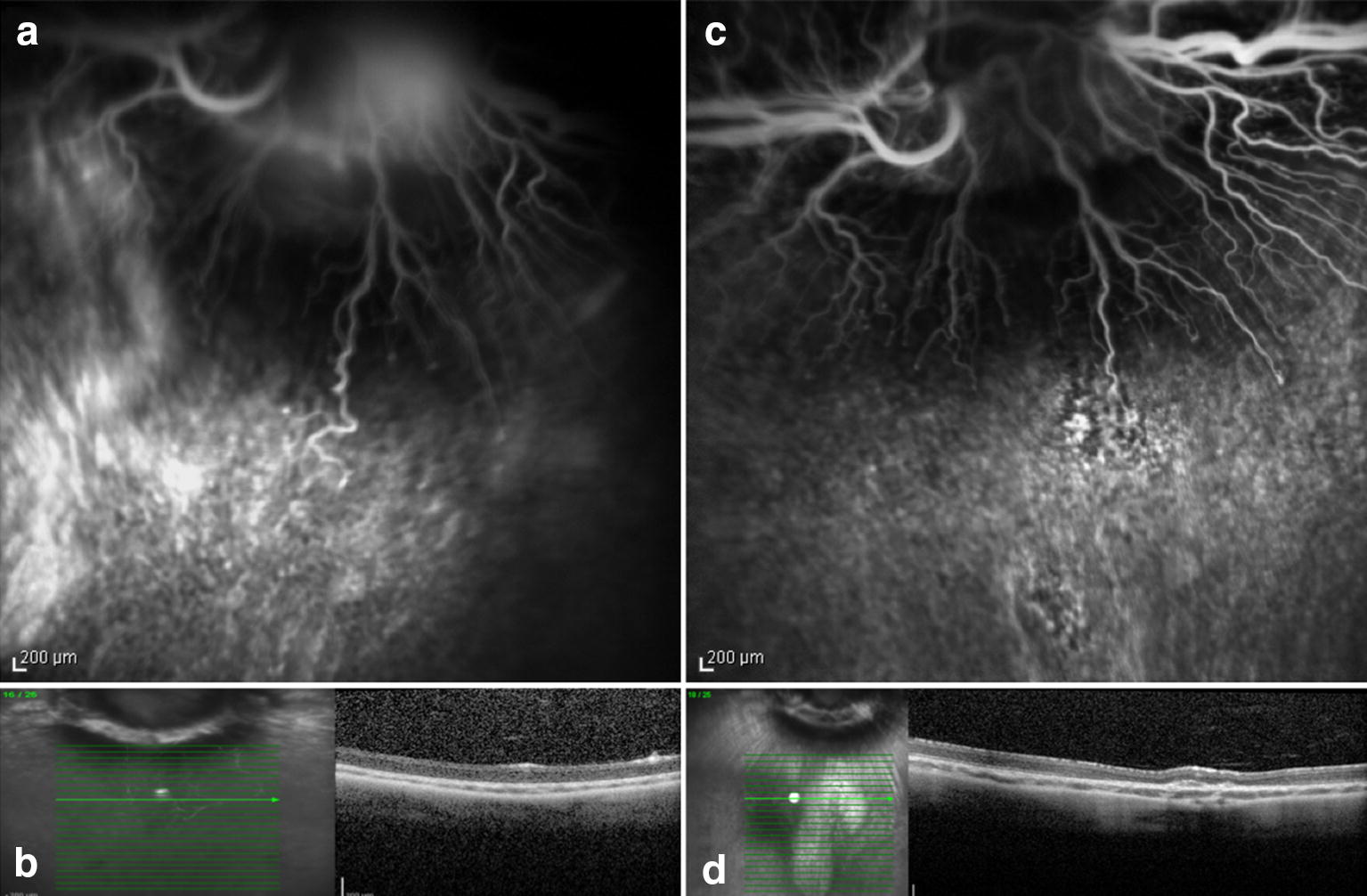
Preoperative (**a** and **b**) and seven days postoperative (**c** and **d**) fluorescein angiography and OCT. Rabbit (no. 6) exposed to 435 nm illumination after systemic administration of lutein. Postoperative hyperfluorescence (**c**) corresponds to the exposed area and increased reflective of the inner and outer layers of the retina seen on OCT (**d**)

**Fig. 5:**
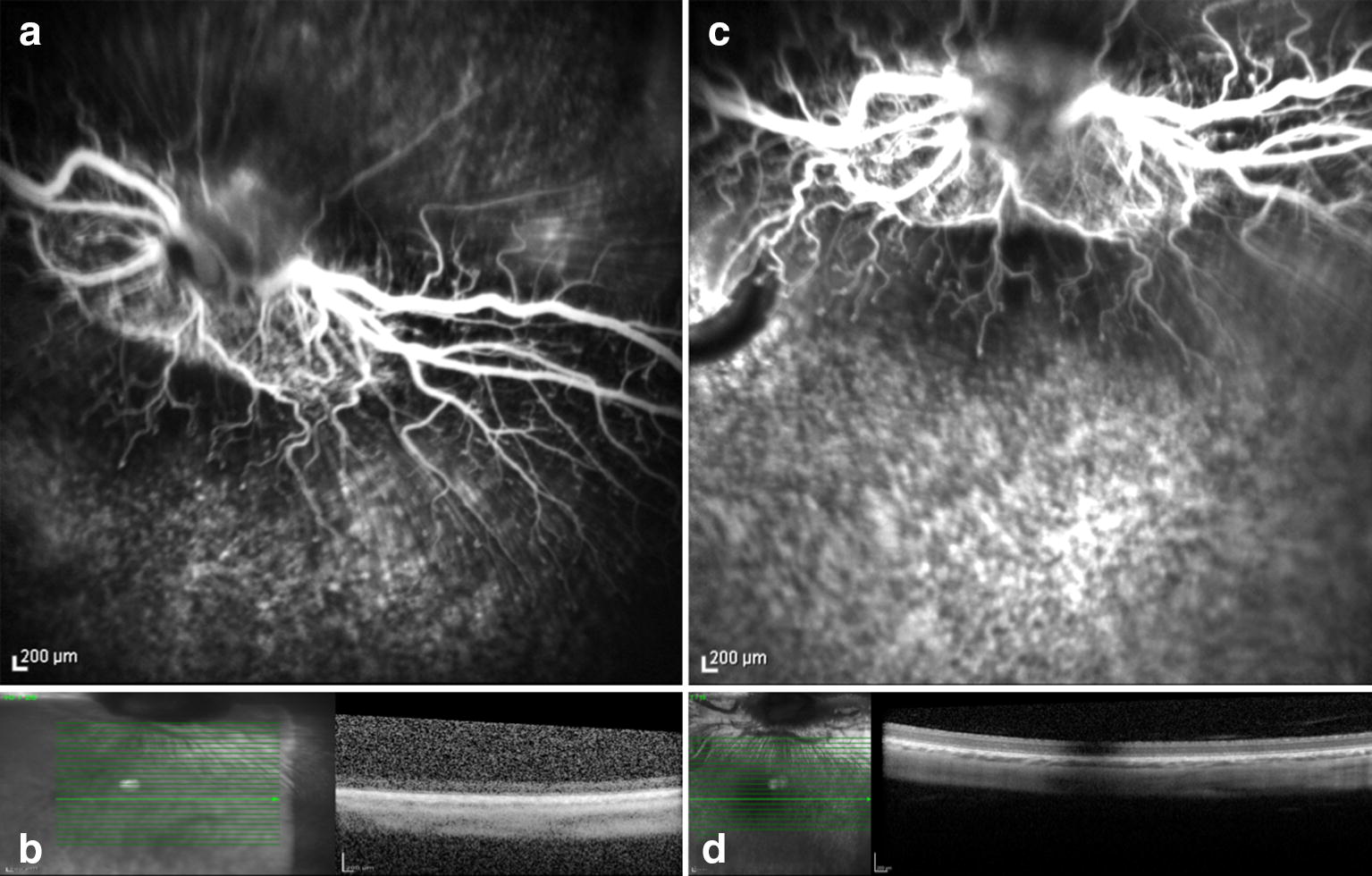
Preoperative (**a** and **b**) and seven days postoperative (**c** and **d**) fluorescein angiography and OCT. Rabbit (no. 8) exposed to 435 nm illumination after systemic administration of lutein. Postoperative hyperfluorescence (**c**) corresponds to the exposed area and increased reflective of the inner and outer layers of the retina seen on OCT (**d**)

**Fig. 6:**
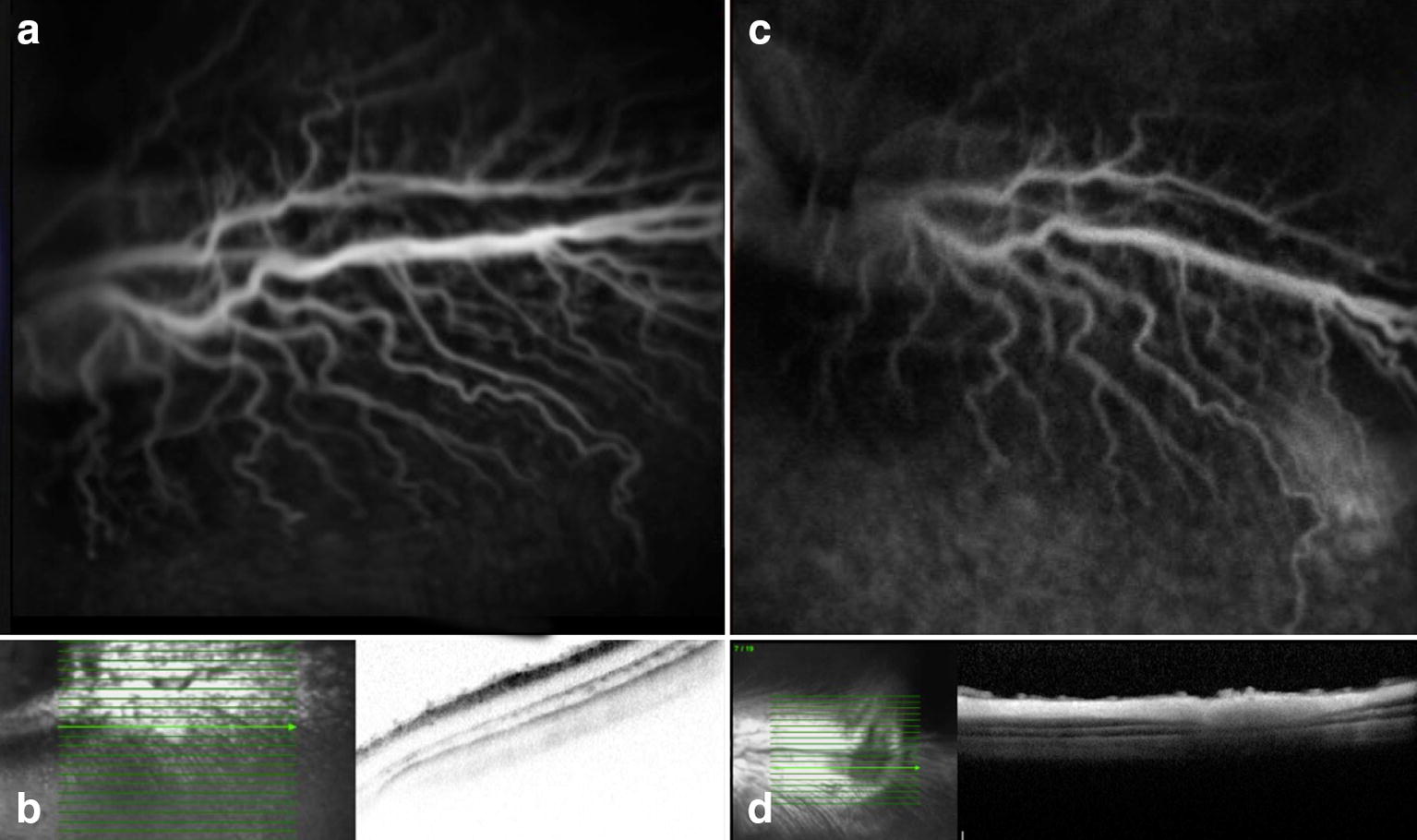
Preoperative (**a** and **b**) and seven days postoperative (**c** and **d**) fluorescein angiography and OCT. Rabbit (no. 12) exposed to light 420 nm without systemic administration of lutein. Postoperative hyperfluorescence (**c**) corresponds to the exposed area and increased reflective of the inner and outer layers of the retina seen on OCT (**d**)

**Fig. 7:**
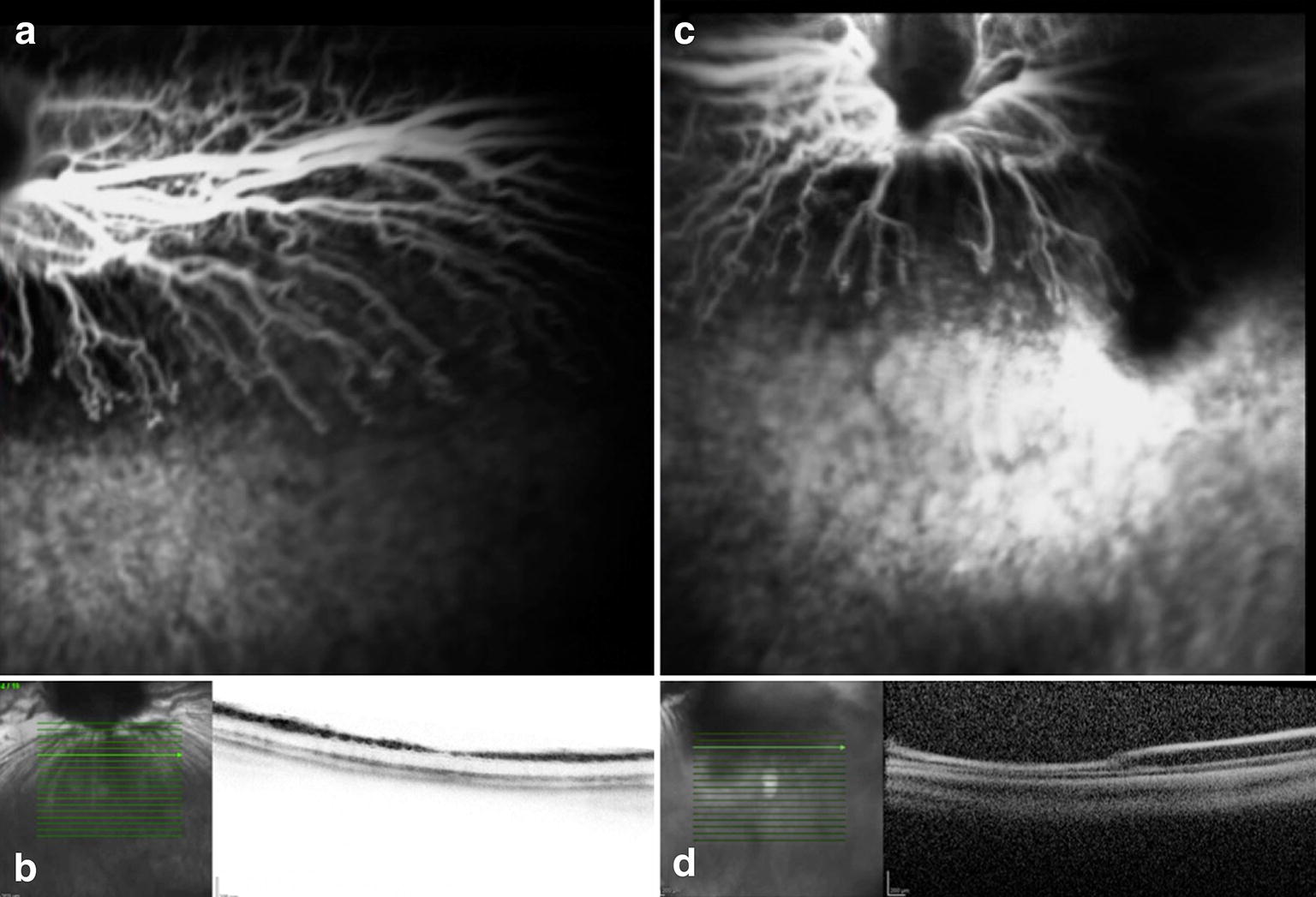
Preoperative (**a** and **b**) and seven days postoperative (**c** and **d**) fluorescein angiography and OCT. Rabbit (no.16) exposed to 435 nm illumination without the administration of lutein. Postoperative hyperfluorescence (**c**) corresponds to the exposed area and increased reflective of the inner and outer layers of the retina with tissue thinning evidenced by examining OCT (**d**)

All animals submitted to the experiment presented some degree of phototoxicity regardless of the wavelength filter and use of systemic lutein. Retinal damage was evident as the FA presented hyperfluorescence in the area of light exposure, and the OCT depicted increased reflective areas of the inner and outer retina layers, and RPE (Tables 1 and 2). We believe that the damage occurred due to the RPE absorption of the wavelength irradiance and possible increase in tissue temperature secondary light exposure (Figs. 2, 3, 4, 5, 6 and 7). The ERG examination showed a significant a and b-waves amplitude decrease (Figs. 1, 2, 3 and 4).

**Table 1:** Rabbits exposed to 420-nm filter wavelength irradiance

Rabbits	Lesion seen on FA	Lesion seen on OCT	Lutein
1	Yes	Yes	Yes
2	Yes	Yes	Yes
3	Yes	Yes	Yes
4	Yes	Yes	Yes
5	Yes	Yes	Yes
11	Yes	Yes	No
12	Yes	Yes	No
13	Yes	Yes	No
14	Yes	Yes	No
15	Yes	Yes	No

**Table 2:** Rabbits exposed to 435-nm-wavelength irradiance

Rabbits	Lesion seen on FA	Lesion seen on OCT	Lutein
6	Yes	Yes	Yes
7	Yes	Yes	Yes
8	Yes	Yes	Yes
9	Yes	Yes	Yes
10	Yes	Yes	Yes
16	Yes	Yes	No
17	Yes	Yes	No
18	Yes	Yes	No
19	Yes	Yes	No
20	Yes	Yes	No

**Fig. 8:**
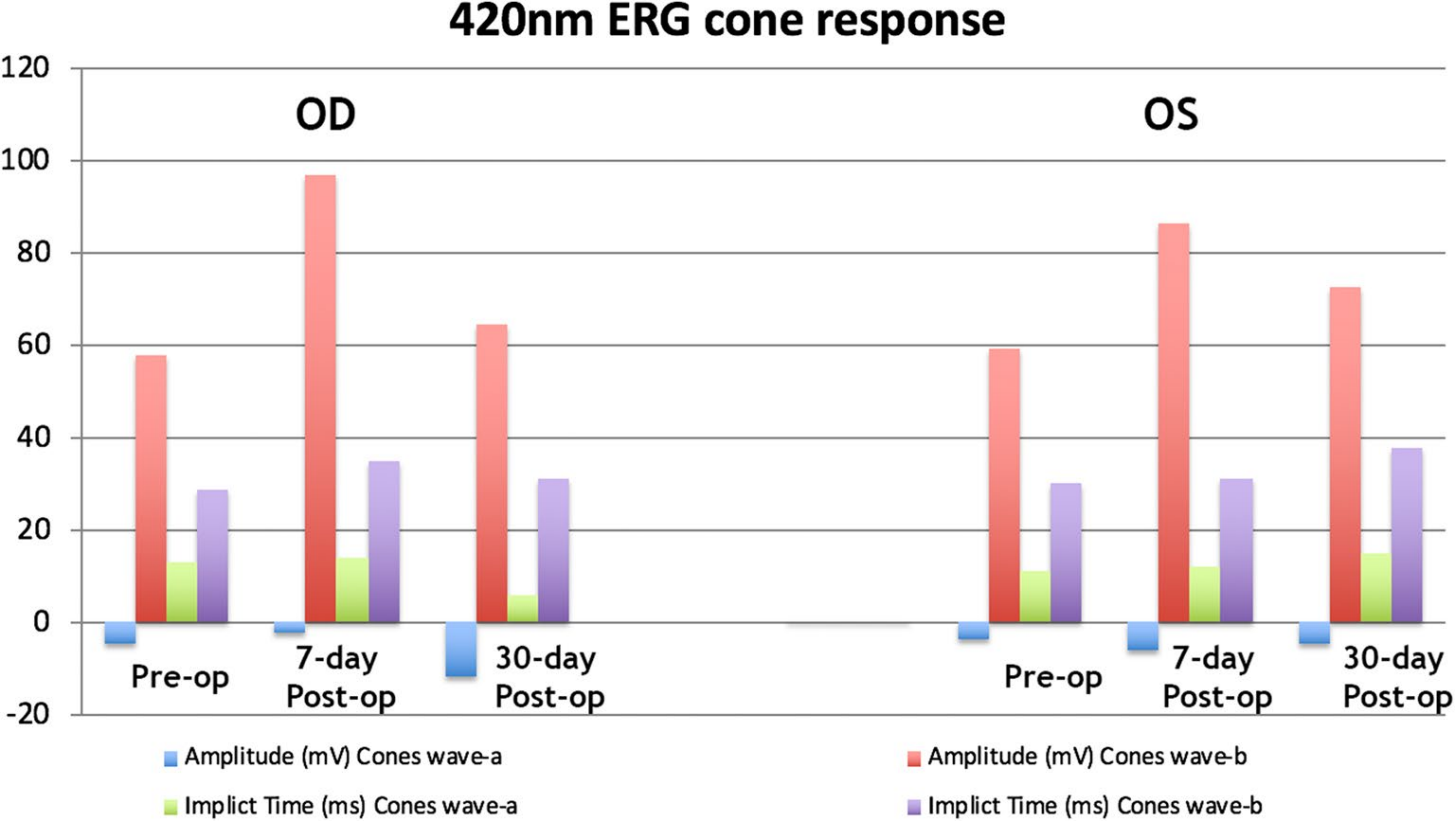
Preoperative and postoperative ERG mean values from the animals exposed to 420 nm wavelength light after systemic administration of lutein. Diffuse reduction of the a and b-wave amplitudes

**Fig. 9:**
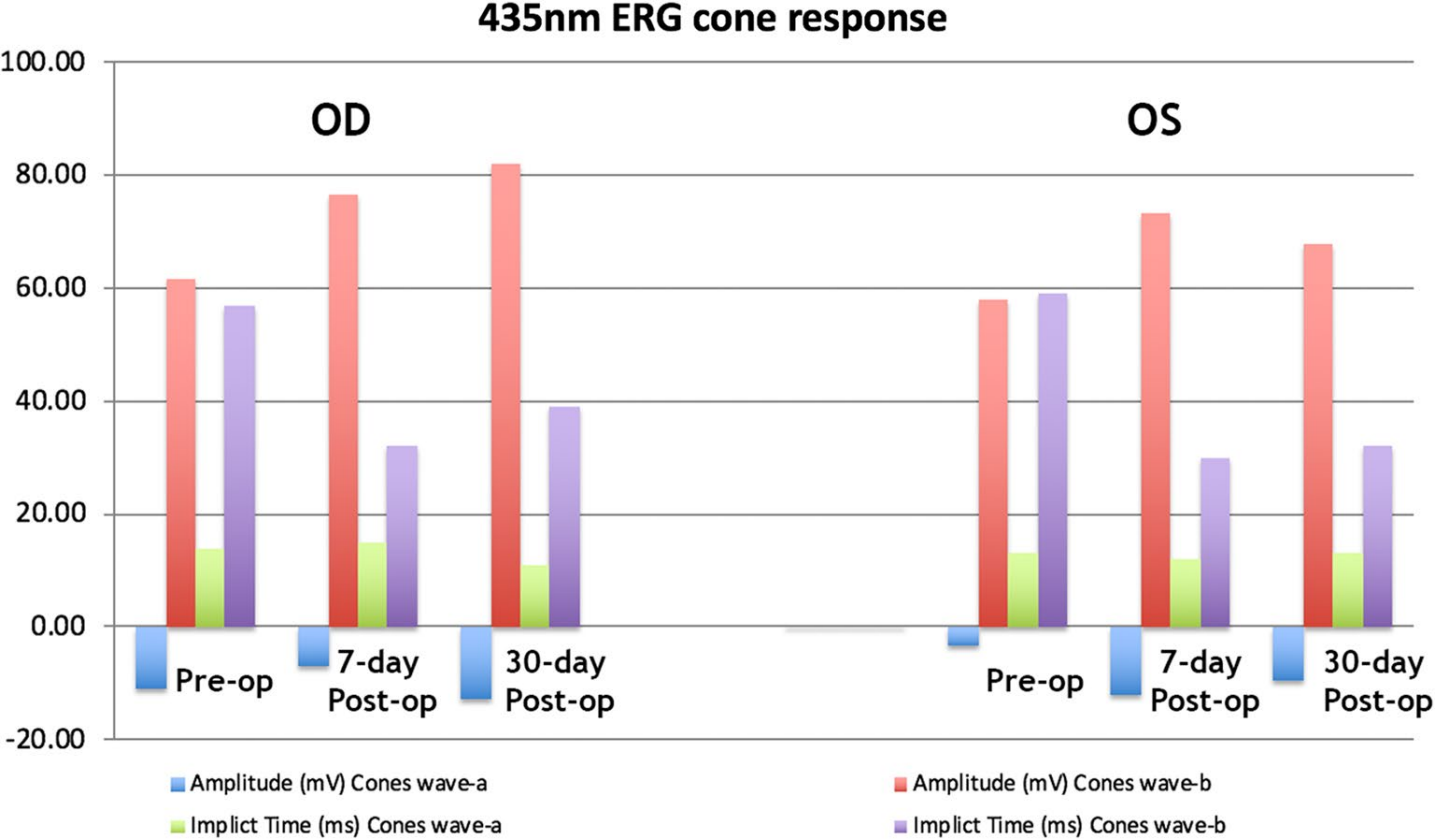
Preoperative and postoperative ERG mean values from the animals exposed to 435 nm wavelength light after systemic administration of lutein. Diffuse reduction of the a and b-wave amplitudes

The FA and OCT from the subgroup exposed to the two different wavelengths after systemic administration of lutein are presented on Fig. 2, 3, 4 and 5. The ERG from the same group is shown on Figs. 8 and 9.

**Fig. 10:**
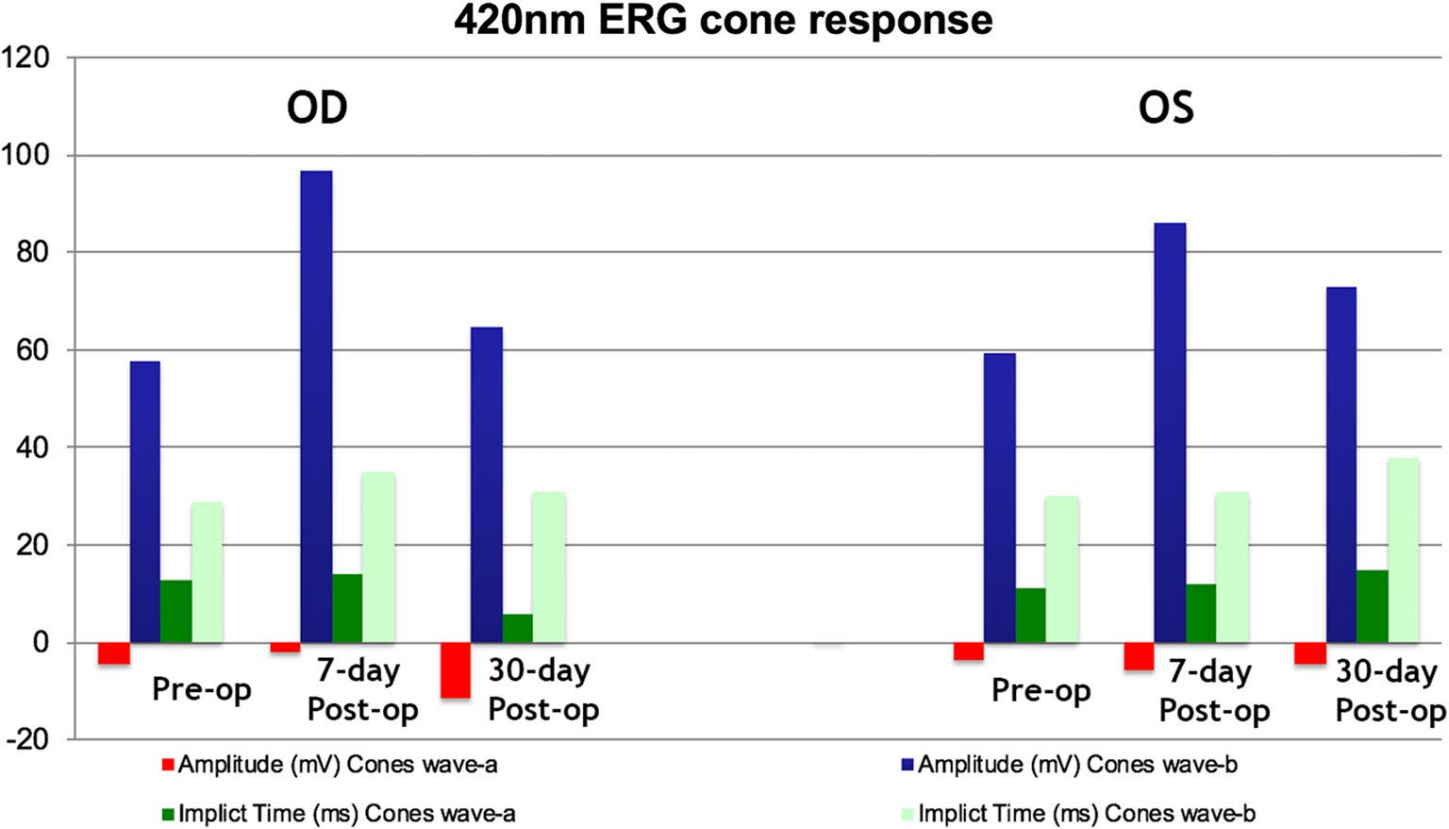
Preoperative and postoperative ERG mean values from the animals exposed to 420 nm wavelength light without systemic administration of lutein. Diffuse reduction of the a and b-wave amplitudes

**Fig. 11:**
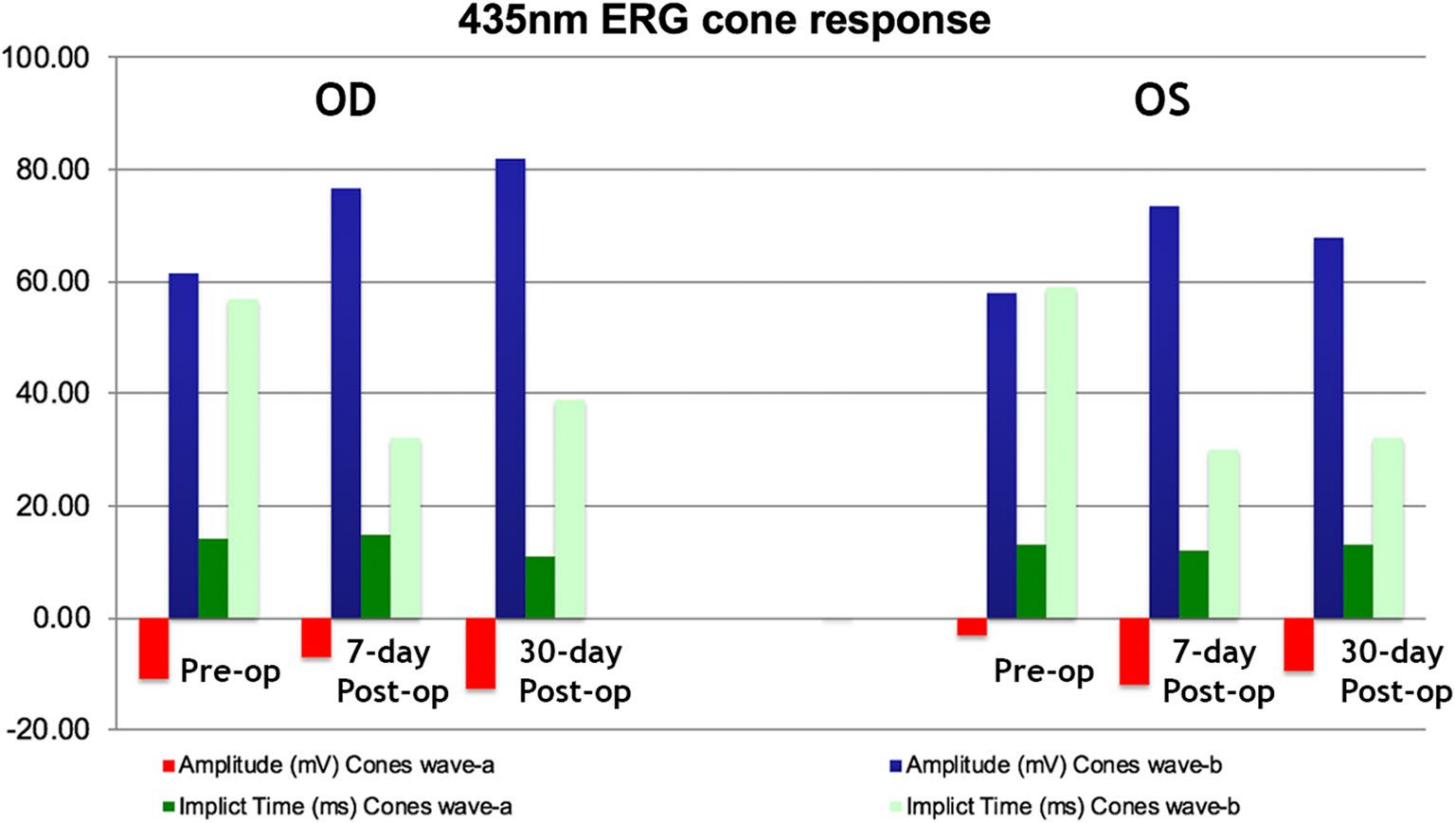
Preoperative and postoperative ERG mean values from the animals exposed to 435 nm wavelength light without systemic administration of lutein. Diffuse reduction of the a and b-wave amplitudes

The FA and OCT from the subgroup exposed to the two different wavelengths without systemic administration of lutein (control groups) are presented on Figs. 6 and 7. The ERG from the same group is shown on the Figs. 10 and 11.

## Discussion

The modern pars plana vitrectomy surgery evolved with increasing gauge and speed of cutting tips. Another crucial factor for surgical success is the better illumination during the surgery, and decrease in tissue damage secondary to light exposure irradiation of the retina and EPR, offered by new light sources such as LED and Xenon [3]. However, improvement in visualization by these new light sources can be associated to greater retinal damage due to the photochemical toxicity of short wavelength (blue and violet). In addition, the RPE may absorb the light, generating heat, thus causing thermal damage. Also, smaller caliber probes can concentrate energy in a specific retina area, since the light is dissipated in proportion to the size of the endoillumination tip. A practical example is a 23-gauge probe, which irradiates a larger area than a 25-gauge probe, dissipating the amount of radiated energy in a larger area, thus, being less harmful.

The photochemical toxicity occurs when the eye is exposed to radiation from shorter wavelengths of visible light (blue and violet), which become more harmful with wavelengths between 380 and 550 nm [5]. The thermal damage is caused by light sources with high-intensity irradiation/irradiance such as laser or xenon, causing the heating of tissues that absorb the radiation. This mechanism does not comply with the principle of reciprocity between the amount of radiation and the duration of exposure; it depends on the conduction of heat by the irradiated tissue. When the target is small, it will cool faster and further irradiation to cause thermal injury is necessary [6].

The macular pigment also functions as a filter that absorbs short wavelengths of visible light, reducing chromatic aberration and dispersion in the fovea. Filtration of short wave helps in preventing photochemical damage to cones and the RPE. The Lutein and Zeaxanthin are present in the photoreceptors axons, pigment in the fovea area and adjacent layers; and theoretically may also have an anti-inflammatory effect [7–15]. In this study, the 420 nm and 435 nm-wavelengths generated damage in both neurosensory retina as well as in the RPE, supporting the literature concern about the use of lighting sources with a wavelength close to ultraviolet waves [16, 17].

In all studied subjects there was evidence of the presence of phototoxicity due to the irradiance of the light sources in simulate vitreoretinal macular surgery. The use of lutein as a protective factor does not prevent the onset of phototoxicity characteristic changes observed in FA and OCT. The anatomical lesions were well evidenced in the examination with FA as they presented as hyperfluorescence, and in OCT as increased reflective of the neurosensory retina and thickening of the line that corresponds to the retinal pigment epithelium. In summary, the FA and OCT findings showed that the EPR plays an important role in generating retinal damage from the thermal and photochemical point of view. All lesions were present in all the wavelengths filters as seen in the figures described in this paper.

The preliminary results shows that the model in pigmented rabbits were suitable for evaluation the retinal damage using FA examination and OCT. Factors such as exposure time, distance from the lighting, wavelength, illumination caliber, can be related to injuries and functional retinal changes.

This study has limitations due to the use of pigmented rabbits. The highly pigmented RPE absorbs the light and can increase tissue temperature, thus contributing to the increase of lesions presented in the results. Future designs using albino rabbits are required to get more accurate results for the phototoxicity wavelength. Another limitation from this study is due to the OCT model used in the experiment, since the absence of “*en face*” image limits the quantitative assessment of the damage of each retina layer.

In conclusion, the use of systemic administration of lutein showed no benefit to avoiding retinal phototoxicity generate to xenon light source using 435 nm and 420 nm filters.

## Abbreviations

ARVO: Association for Research in Vision and Ophthalmology
ERG: electroretinography
FA: fluorescein angiography
ISCEV: International Society for Clinical Electrophysiology of Vision
MS: milliseconds
OCT: optical coherence tomography
RPE: retinal pigment epithelium
µV: microvolts
